# CAR-T cell therapy for pediatric solid tumors: armored CAR-T cells and beyond

**DOI:** 10.1007/s10555-026-10317-2

**Published:** 2026-03-02

**Authors:** Jeremy Wells, Ajay Gupta

**Affiliations:** 1 Jacobs School of Medicine and Biomedical Sciences, Buffalo, NY USA; 2 Department of Pediatric Oncology, Roswell Park Cancer Institute, Buffalo, NY USA

**Keywords:** CAR-T, Pediatric, Solid, Tumor, Immunotherapy

## Abstract

CAR-T cell therapy, which uses endogenous T cells engineered to target specific cancer antigens, is one of the most promising recent developments in the treatment of hematologic malignancies in both children and adults. CAR-T cells have shown tremendous success in treating B-cell lymphoma, acute lymphoblastic leukemia, and multiple myeloma, and they are currently FDA-approved for the treatment of six hematologic malignancies. Its success in solid tumors has been more modest, which has been attributed to several factors including the hostile tumor microenvironment (TME), poor persistence of CAR-T cells, and difficulty directing CAR-T cells towards solid tumors. Armored CAR-T cells, which modify the TME via secreted cytokines, have shown early success in the treatment of solid pediatric malignancies. We review recent trials of CAR-T cells to treat common pediatric solid malignancies, including Ewing sarcoma, osteosarcoma, neuroblastoma, diffuse intrinsic pontine glioma, rhabdomyosarcoma, Wilms tumor, and retinoblastoma. We focused particularly on armored CAR-T cells where applicable. Armored CAR-T cells have been utilized to target a variety of tumor-associated antigens on pediatric solid tumors with early successes both *in vivo* and *in vitro*, and innovative approaches for addressing their limitations are rapidly being developed.

## Introduction

CAR-T cells represent a burgeoning field in oncology and are a promising treatment option for a variety of malignancies. CAR-T therapy relies on endogenous T cells which have been engineered *ex vivo* to target a particular antigen present on cancer cells. The portion of the CAR-T cells which directly interacts with the antigen is known as the ligand-binding domain. Typically, this takes the form of a single-chain variable fragment (scFv), which is composed of the variable regions of the light and heavy chains of an immunoglobulin [1]. In other instances, nanobodies, native receptors, and ligands to cognate receptors have been used as ligand-binding domains [1]. A transmembrane component binds the ligand-binding domain to the T cell and transduces the signal to an intracellular signal transduction portion containing an immune-receptor tyrosine-based activation motif (ITAM), which leads to downstream signaling and T cell activation. Additionally, CAR-T cell activation is MHC-independent, which circumvents the loss of MHC-associated antigen presentation that occurs in cancer cells. The first three generations of CARs can be distinguished by the presence or absence of co-stimulatory domains. First-generation CAR-T cells contain only the intracellular signaling domain CD3ζ. Second-generation CAR-T cells contain one co-stimulatory domain (CD28 or 4-1BB) bound to CD3ζ, and third-generation CARs contain two co-stimulatory domains (CD28 and 4-1BB) bound to CD3ζ [2]. CAR-T cells provide an advantage over traditional chemotherapy and radiotherapy in their ability to target specific antigens and discriminate between malignant and nonmalignant tissue. Ideally, this would reduce the need for chemotherapy and its associated systemic toxicities.

CAR-T therapy has proven very successful in treating a variety of cancers, most notably relapsed and refractory B cell malignancies such as B-cell acute lymphocytic leukemia (B-ALL), non-Hodgkins lymphoma (NHL), and multiple myeloma (MM) [3]. The success of CAR-T therapy, however, has been largely confined to hematological malignancies, with limited success in solid tumors [4, 5]. Whereas CAR-T cells used to treat hematologic malignancies are infused directly into the blood, CAR-T cells must penetrate solid tumors and their surrounding environments in order to be effective. The poor responses to CAR T cell therapy seen in solid tumors are multifactorial and may stem from several interrelated factors. Identifying highly specific tumor-associated antigens with low expression on normal tissue has proven difficult but is crucial for mitigating damage to healthy cells. The heterogeneous expression of tumor antigens among solid tumors can lead to antigen escape, a process by which tumor cells can evade targeting by immunotherapy [6]. Perhaps the most crucial obstacle in the treatment of solid tumors is the presence of a the immunosuppressive tumor microenvironment (TME) that facilitates tumor growth and inhibits the effectiveness of immunotherapeutic modalities like CAR-T cells. The TME is composed of dense stroma and immune cells, including cancer-associated fibroblasts (CAFs), tumor-associated macrophages (TAMs), regulatory T cells (Tregs), and myeloid-derived suppressor cells (MDSCs) that prevent effective T cell engagement and cause increased T cell exhaustion [7].

These immunosuppressive cells and the cytokines they secrete—including IL-10, TGF-β, and IL-4—can inhibit CAR-T cell antitumor activity and contribute to cancer proliferation. TAMs, chiefly composed of the alternatively activated M2 phenotype, inhibit tumoricidal responses, promote angiogenesis, and prevent effector T cells from entering the tumor [7–9]. Their ability to promote IL-8 secretion by Tregs enhances TGF-β production and dampens antitumor activity. In addition to utilizing the aforementioned methods, MDSCs produce matrix metalloproteinase-9 and remodel the extracellular matrix, making it more hospitable for tumor growth [7].

**Fig. 1:**
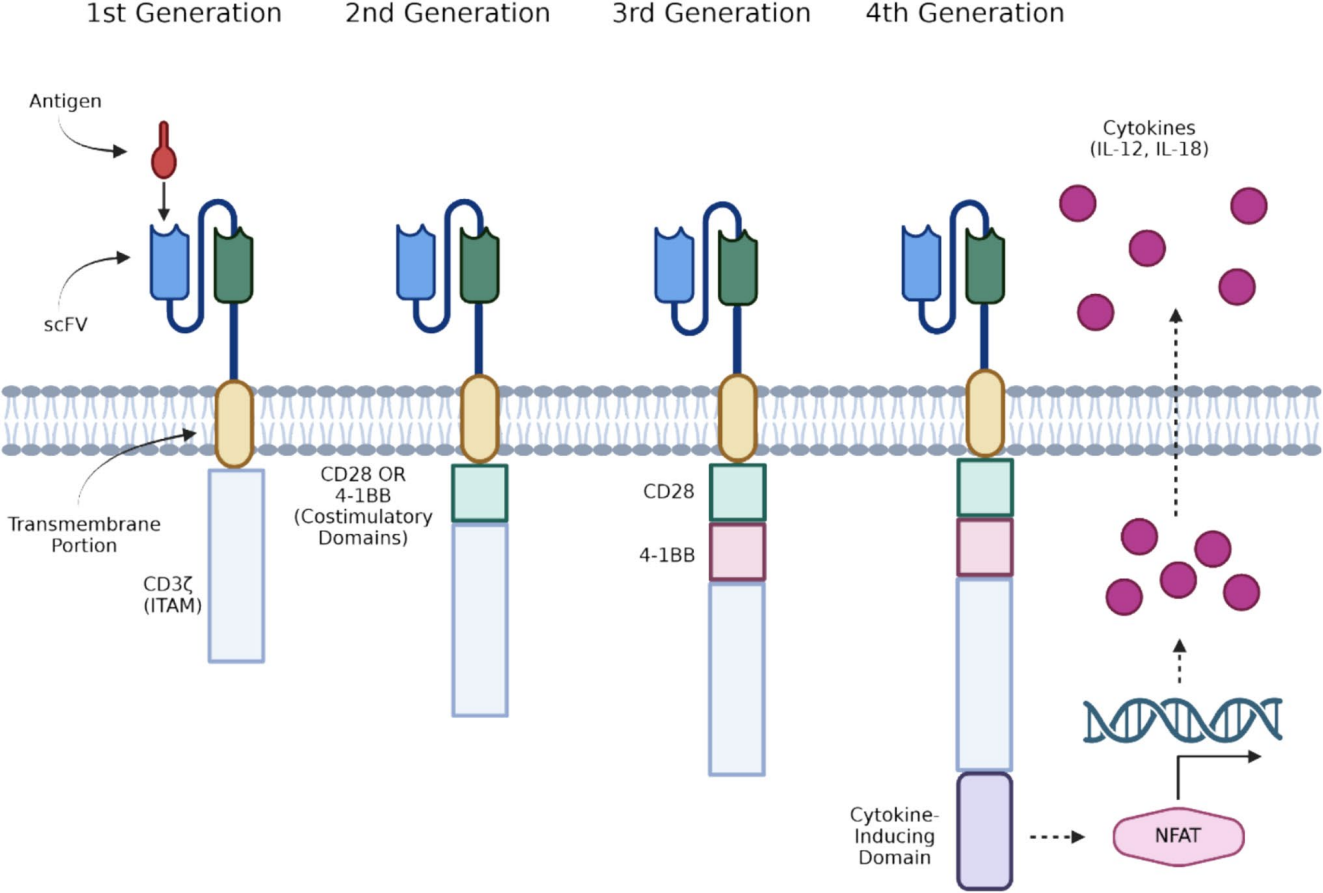
The structures of CAR-T cell generations one through four. First-generation CAR-T cells combined an antigen-recognizing domain, a transmembrane portion, and an ITAM. Second-generation CAR-T cells added either a CD28 or 4-1BB costimulatory domain, while third-generation CAR-T cells utilized both costimulatory domains. Fourth-generation CAR-T cells have an additional cytokine-inducing domain which leads to the transcription of cytokines after the antigen-recognizing domain is stimulated. Created in BioRender. Genetics, C. (2026) https://BioRender.com/8453mc1

To overcome the immunosuppressive TME, fourth-generation “armored” CAR-T cells have been developed, which combine a traditional antigen-recognizing domain with elements which modify and interact with the TME when activated. TRUCK (T cells redirected towards universal cytokine killing) CAR-T cells are a subset of armored CAR-T cells which secrete cytokines to counteract the immunosuppressive properties of the TME (Fig. 1) [8]. Additionally, other subsets of armored CAR-T cells function by modifying the way in which cells respond to these immunosuppressive TME. One study combined an IL-7 receptor domain with an IL-2 intracellular signaling domain so that an immunosuppressive IL-7 signal was transduced into pro-inflammatory downstream IL-2 signaling [10].

CAR-T therapy has significant implications in the treatment of pediatric malignancies. Blood cancers such as leukemia represent the most common variety of pediatric cancer, and CAR-T cell treatments have proven successful in treating these cancers. Kymirah (tisagenlecleucel), the first FDA-approved CAR-T cell treatment, is a CD-19-directed CAR-T cell approved for the treatment of relapsed or refractory B-ALL in both children and adults. While ALL is the most common single type of malignancy in children, solid tumors still comprise 60% of all pediatric malignant neoplasms [11]. The most common solid tumors in children include CNS tumors, embryonal tumors, and sarcomas [11]. Despite the obstacles that CAR-T therapy has encountered in treating solid tumors, armored CAR-T cells represent an encouraging treatment option. This review will focus on the treatment of the most common subtypes of solid tumors in children with the use of CAR-T cells, with particular attention paid to armored CAR-T cells where applicable.

## Methods

A literature review of preclinical and clinical studies of CAR-T cells for the most common pediatric solid tumors was conducted between February 2025 and December 2025 using the NCBI PubMed database, Embase, and conference abstracts from AACR, ASTCT, ASCO, ESMO, and SIOP. Articles which involved CAR-T cells being tested in one of the most common types of pediatric solid tumors, including neuroblastoma (NB), osteosarcoma (OS), Ewing sarcoma (ES), rhabdomyosarcoma (RMS), diffuse intrinsic pontine glioma/diffuse midline glioma (DMG/DIPG), retinoblastoma (RB), and Wilms tumor (WT) in a preclinical or clinical setting were included. Particular attention was paid to CAR-T cells tested in pediatric tumors which were “armored” with the addition of cytokines or immune modulators to combat the hostile TME (Tables 1, 2). A search of the ClinicTrials.gov database was also performed of all active and completed clinical trials involving CAR-T cells in one of the aforementioned pediatric malignancies.

**Table 1:** Preclinical and clinical results from studies using fourth-generation armored CAR-T cells with their associated target antigens, armoring cytokines or receptors, and tumor type they were studied in

Preclinical			
Antigen target(s)	Armoring element(s)	Tumor studied	Result summary
GD2	IL-15	NB	Superior antitumor activity and survival; IL-15 produced cells with “memory” and “stem cell-like” properties [12]
GD2	IL-18	NB	Enhanced cytotoxic effects *in vitro*; mice treated with armored GD2 CAR-T cells survived until the end of the 24-day observation period [13]
GD2	IL-18	NB	High levels of cytokine release compared to background levels and efficacious killing of GD2-positive NB cells *in vitro *[14]
GD2	IL-7, CCR2b	NB	In a murine model, GD2 CAR-T cells co-expressing IL-7 and CCL2b inhibited tumor growth, whereas non-armored CAR-T cells did not [15]
GD2	P40 (IL-23 subunit)	NB	In a metastatic NB model in mice, these cells exhibited superior antitumor activity without side effects compared to CAR-T cells engineered with IL-18 or IL-15, which produced weight loss in the mice [16]
GD2	IL-15	RB	In the cohort of mice treated with the IL-15-releasing CAR-T cells, there was significant antitumor activity, with 60% of these mice remaining tumor free up to day 70 [17]
B7-H3	CXCR2 (IL-8R)	OS	Overexpression of CXCR2 significantly enhanced homing into IL-8 expressing tumors such as OS [18]
B7-H3	CXCR2, CXCR6	OS	These modifications enhanced CAR-T cell homing towards OS cells both *in vitro* and *in vivo*, including in a metastatic murine OS model [19]
B7-H3	CSTR	OS, ES, NB	In orthotopic models of NB, ES, and OS, these CAR-T cells were effective at penetrating tumors and controlling disease, while unmodified B7-H3 CAR-Ts failed [20]
B7-H3	CXCR3	DIPG/DMG	In an orthotopic mouse model of DIPG, CAR-T cells modified with CXCR3 maintained complete tumor regression, whereas four of five mice treated with unmodified B7-H3 CAR-T cells experienced rebound of tumor signals [21]
NKG2D	CXCR5, IL-7	OS	CXCR5/IL-7 CAR-T cells demonstrated superior cytokine production, decreased exhaustion, and superior cytotoxicity against OS in mouse models compared to wild-type CAR-T cells [22]
NKG2D	IL-18	OS	High levels of IL-18 secretion upon antigen challenge and superior antitumor activity compared to non-armored anti-NKGD2 CAR-T cells in a murine model [23]
TNC	IL-18R	OS	Anti-TNC CAR-T cells expressing a constitutively active IL-18R displayed superior antitumor effector function *in vitro* and prolonged mouse survival in a murine OS xenograft compared to non-armored anti-TNC CAR-T cells [24]
GPC2	IL-15, IL-21	NB	Enhanced NB cell killing relative to control anti-GPC2 CAR-T cells [25]
Clinical			
Antigen target(s)	Armoring element(s)	Tumor studied	Result summary
GD2	IL-15	NB	The objective response rate was 25% (3/12), with one complete response and two partial responses [26]
GD2	IL-7R	DIPG/DMG	Patients receiving these CAR-T cells showed a temporary improvement in neurologic deficits, and seven of eight were eligible for additional treatment cycles [27]
GPC3	IL-15	WT, RMS, HCC	The patient with WT experienced progressive disease, while the patient with RMS exhibited a partial response to treatment [28]

**Table 2:** List of completed, active, and recruiting clinical trials for CAR-T cells in pediatric solid tumors organized by target antigen. Certain trials specify inclusion criteria for children with particular malignancies; others are open to any patient with histologically confirmed expression of a target antigen. For trials with results reported, they are referred to later in the manuscript and referenced by NCT number

Target antigen	Pediatric malignancy	NCT number	Phase	Status	Notes
**GD2**	OS, NB	NCT02107963	1	Completed	
		NCT07211737	1	Not yet recruiting	
		NCT03721068	1	Recruiting	Armored with IL-15 and inducible caspase-9 safety switch
	GD2-positive tumors, NB	NCT01953900	1	Active, not recruiting	GD2 CAR-T cells plus VZV vaccine and lymphodepletion
		NCT03373097	1, 2	Active, not recruiting	
		NCT03373097	1, 2	Active, not recruiting	
	NB, sarcomas, uveal melanoma, breast cancer, or other GD2-positive cancers	NCT03635632	1	Active, not recruiting	Armored with C7R
	NB	NCT06684639	1	Recruiting	
		NCT05990751	1	Recruiting	
		NCT02919046	1	Unknown	
		NCT03294954	1	Recruiting	
		NCT01822652	1	Active, not recruiting	
		NCT01460901	1	Completed	
		NCT02765243	1	Completed	
		NCT02761915	1	Completed	
		NCT02439788	1	Withdrawn	
		NCT00085930	1	Active, not recruiting	
	DIPG/DMG, medulloblastoma, high-grade glioma	NCT05298995	1	Recruiting	
	DIPG/DMG, GD2-expression CNS tumors	NCT04099797	1	Recruiting	Armored with C7R
	DIPG/DMG	NCT04196413	1	Recruiting	
	DIPG/DMG	NCT05544526	1	Recruiting	
**B7-H3**	B7-H3-positive sarcomas	NCT07222735	1	Recruiting	CAR-T cells plus hypofractionated radiation
	B7-H3-positive tumors	NCT07052383	1	Not yet recruiting	
		NCT04483778	1	Active, not recruiting	
		NCT04897321	1	Recruiting	
	OS, NB, gastric, lung	NCT04864821	1	Unknown	
	NB, OS, ES, WT, soft tissue sarcoma	NCT06500819	1	Recruiting	
	RMS, ES, NB, WT	NCT07172958	1	Not yet recruiting	
	NB	NCT05562024	1	Recruiting	
	DIPG/DMG	NCT06221553	1	Recruiting	B7-H3 CAR-T cells with IL7Ra signaling domain
		NCT04185038	1	Recruiting	
	DIPG/DMG or non-brainstem CNS tumor	NCT05835687	1	Recruiting	
**GPC3**	GPC3-positive solid tumors	NCT04377932	1	Active, not recruiting	Armored with IL-15
		NCT04715191	1	Recruiting	Armored with IL-15 and IL-21
		NCT07148050	1	Not yet recruiting	Armored with IL-15 and IL-21
**HER2**	HER2-positive sarcoma	NCT04995003	1	Recruiting	HER2 CAR-T cells plus immune checkpoint inhibitor
**HER2, CD146**	Soft tissue or bone sarcoma	NCT07066982	1, 2	Recruiting	
**HER2, GD2, PSMA, CD276**	Sarcomas with HER2, GD2, PSMA, or CD276 positivity	NCT04433221	1, 2	Unknown	
**GD2, PSMA, CD276**	NB	NCT04637503	1, 2	Unknown	
**GD2/B7-H3**	NB	NCT06836505	1, 2	Recruiting	
**IM83**	OS	NCT06412458	1	Not yet recruiting	
**UB-TT170**	OS	NCT05312411	1	Active, not recruiting	
**FOLR1**	OS	NCT07227571	1	Not yet recruiting	
**Sarcoma-specific (patient-dependent)**	OS	NCT03356782	1, 2	Unknown	
**EGFR806**	Non-CNS solid tumors expressing EGFR	NCT03618381	1	Active, not recruiting	
**PHOX2B**	NB	NCT07007117	1	Recruiting	
**GPC2**	NB, RB	NCT05650749	1	Recruiting	
**CD171/L1CAM**	NB	NCT02311621	1	Active, not recruiting	
**hALK**	NB	NCT06803875	1, 2	Recruiting	
**FGFR4**	RMS	NCT06865664	1	Recruiting	
**B7-H3, EGFR806, HER2, IL-13 zetakine**	DIPG/DMG	NCT05768880	1	Active, not recruiting	

## Results

### Osteosarcoma

Osteosarcoma (OS) is the most common primary bone tumor in children and adolescents, with an incidence of 5 cases per million in children and adolescents up to 19 years [11]. It is a malignant neoplasm of osteoid-secreting mesenchymal stem cells which typically arises in the metaphysis of long bones. The standard of care includes surgical resection and chemotherapy with methotrexate, cisplatin, and doxorubicin [11]. While cure rates for nonmetastatic osteosarcoma have improved to greater than 60%, cure rates for patients with metastatic disease are as low as 19% [29, 30]. The pleomorphism of osteosarcoma cells presents a challenge regarding chemotherapy resistance, as 15–20% of patients with metastatic OS are resistant to first-line chemotherapeutics [31]. This pleomorphism has also made engineering a CAR-T antigen that precisely targets OS difficult.

The diganglioside GD2 has emerged as a target for cancer immunotherapy due to its relatively restricted expression to solid tumors such as sarcomas [32]. It was found to be present on 55% of primary sarcomas, with the highest expression among OS and alveolar rhabdomyosarcoma [32]. While GD2-directed CAR-T cells demonstrated potent *in vitro* and *in vivo* activity against OS, tumor-derived G-CSF limited the effectiveness of GD2-directed CAR-T cells and contributed to the immunosuppressive TME by driving the expansion of myeloid-derived suppressor cells (MDSCs) [32]. Due to the immunosuppressive effects of G-CSF and its ubiquity in the OS TME, its inhibition remains a potential target for the augmentation of CAR-T therapy. A study by Sterner et al. demonstrated that the neutralization of G-CSF with Lenzilumab enhanced CD-19 directed CAR-T cell proliferation in leukemia and led to a reduction in cytokine release syndrome (CRS), which they hypothesized was due to G-CSF’s role in leukemia cell proliferation [33].

In an abstract, Ramakrishna et al. reported the initial results of a phase I trial of GD2-CARTs in children and young adults with GD2-positive solid tumors, including OS and neuroblastoma (NCT02107963). Thirteen patients were infused, with two cases of grade-1 cytokine release syndrome and no neurological or dose-limiting toxicity [34]. On day 28 after infusion, 10 of 13 patients had stable disease and three had progressive disease, with all patients eventually progressing [34]. Following these results, they analyzed the immune profiles of the patients in order to determine factors influencing adequate CAR-T cell expansion, concluding that proliferation of the GD2-CARTs was correlated with a larger baseline population of naïve and central memory T cells [34].

Human epidermal growth factor receptor 2 (HER2) is expressed on a wide range of cancers, including lung, ovary, prostate, brain, and sarcomas [35]. Most sarcomas, however, express HER2 at levels which are too low to allow for effective targeting by HER-directed monoclonal antibodies (mAbs) such as trastuzumab. Ahmed et al. reported the results of a phase I/II clinical trial of HER2-CARTs in patients with HER2-positive tumors (NCT00902044). Nineteen patients had been enrolled, 16 of whom had osteosarcoma. The HER2-CARTs persisted for 6 weeks in seven of the nine evaluable patients [36]. Three patients had their tumors removed, and one showed greater than 90% tumor necrosis [36].

Activated leukocyte cell adhesion molecule (ALCAM, CD166) is a membrane glycoprotein that, by binding to CD6, mediates interactions between adjacent leukocytes [37]. In a study by Wang et al., the expression of ALCAM on four OS cell lines ranged from 36.9 to 96.7% [37]. After establishing the high levels of ALCAM in OS cell lines, Wang et al. constructed an orthotopic OS model using immunodeficient mice and tibially implanted OS cells. When anti-ALCAM CAR-T cells were transfused into these mice, there was a significant reduction in tumor weight compared to control [37].

B7-H3 is a cell surface glycoprotein and another promising target antigen for CAR-T cells. While its precise physiological function has not been elucidated, it is believed to function as an immune checkpoint molecule. It is highly expressed among cell surfaces of pediatric solid tumors, including neuroblastoma, rhabdomyosarcoma, Wilms tumor, Ewing sarcoma, osteosarcoma, and diffuse midline glioma, but has limited expression on healthy tissue [38–41]. In addition to being expressed on cancer cells, B7-H3 is also upregulated on component cells of the TME, such as dendritic cells (DCs), tumor-associated macrophages (TAMs), myeloid-derived suppressor cells (MDSCs), neutrophils, and tumor-associated endothelial cells. The immunosuppressive functions of B7-H3 interfere with IFN-γ release and NK cell cytotoxicity, reducing the efficacy of CAR-T treatment [39]. Consequently, CAR-T cells targeting B7-H3 may have the dual function of eliminating tumor cells and peripheral cells contributing to the TME [38, 42]. Upregulation of B7-H3 has also been demonstrated on B7-H3-directed CAR-T cells when co-cultured with antigen-positive solid tumor cells, indicating that CAR-T cell fratricide may be a potential limitation of this therapy [43].

Zhang et al. tested anti-B7-H3 CAR-T cells both *in vitro* and *in vivo* in a murine model. They first confirmed high B7-H3 expression of 73.3% among 60 pathological OS sections [44]. *In vitro*, their CAR-T cells recognized and killed B7-H3-positive OS cells. *In vivo*, these CAR-T cells showed excellent anti-tumor activity in both the high- and low-dose group [44].

Talbot et al. investigated B7-H3 CAR-T cells in a metastatic OS model in which mice were implanted with an OS cell line (LM7) with high B7-H3 expression and monitored for the development of metastases [45]. B7-H3 CARTs demonstrated excellent antitumor activity *in vivo* and prevented the development of pulmonary metastases, which was monitored using firefly luciferase (ffLuc) expressed by the OS cells [45]. In a subsequent study, Talbot et al. harnessed chemokines secreted by OS cells to more precisely direct B7-H3 CAR-T cells to their target tumor. They identified IL-8 and CXCL16 as most highly expressed chemokines on OS samples and modified anti-B7-H3 CAR-T cells with CXCR2 and CXCR6, these chemokines’ cognate receptors. These modifications enhanced CAR-T cell homing towards OS cells both *in vitro* and *in vivo*, including in a metastatic murine OS model [19].

Adeshakin et al. constructed anti-B7-H3 CAR-T cells with a knockout of the reg1 gene, which produces a potent negative immune regulatory molecule known as regnase-1, and tested them in a murine model of OS [46]. Deletion of reg1 improved the function of these CAR-T cells, which were able to promote a more effective proinflammatory landscape compared to nonmodified CAR-T cells [47]. Second-generation B7-H3 CAR-T cells developed by Majzner et al. demonstrated promising activity against OS, ES, and medulloblastoma in an orthotopic murine model [48].

Pinto et al. reported results from the STRIvE-02 clinical trial of B7-H3 CAR-T cells in pediatric patients (NCT04483778). While three patients with OS were enrolled, the study was open to all B7-H3 positive tumors, and patients with Ewing Sarcoma, neuroblastoma, and rhabdomyosarcoma were also included [49]. Nine patients in total were treated at two different dose levels, with three experiencing stable disease and six experiencing progressive disease [49]. A clinical response was noted in one patient after being treated with a second infusion at dose level two [49].

Other tumor antigens are also highly expressed on OS cell lines, such as ALPL-1, an isoform of alkaline phosphatase, and IL-11RA, a receptor for IL-11 [50, 51]. CAR-T cells directed towards these antigens demonstrated preferential targeting of tumor cells without significant systemic toxicity in *in vivo* models of orthotopic primary and metastatic OS [50, 51].

Xin Huang et al. investigated insulin-like growth factor receptor (IGF1R) and tyrosine kinase-like orphan receptor 1 (ROR1) as potential CAR-T targets and determined that both were highly expressed among OS, ES, and RMS cell lines [52]. Transfusion of these CARTs into NSG mice intravenously inoculated with OS cells led to reduced tumor growth and prolonged survival [51].

Shin et al. investigated the feasibility of programmed cell death ligands 1 and 2 (PD-L1, PD-L2) as CAR-T targets by assessing their expression on OS, NB, GBM, and RMS cell lines. They found that PD-L1 and PD-L2 expression were upregulated in OS after stimulation with IFN-γ and TNF-α, while their expression remained low on other cell lines [53]. They then tested PD-1 CAR-T cells against an OS cell line that expresses moderate levels of PD-L1 and observed that the CAR-T cells displayed cytotoxic activity in a dose-dependent manner *in vitro* [53].

Combating the hostile tumor microenvironment remains an obstacle in the treatment of OS, and fourth-generation “armored” CAR-T cells attempt to overcome this by co-expressing or secreting cytokines with antitumor activity. Hui et al. developed an NKG2D-directed CAR-T construct based on previous studies’ success using this ligand against OS cell lines *in vitro* and in murine models and engineered it to co-express IL-7 and CXCR5, a chemokine receptor whose ligand is overexpressed in OS [22, 54, 55]. CXCR5/IL-7 CAR-T cells demonstrated superior cytokine production, decreased exhaustion, and superior cytotoxicity against OS in mouse models compared to wild-type CAR-T cells [56]. An IL-18-armored anti-NKG2D CAR-T engineered by Breman et al. showed high levels of IL-18 secretion upon antigen challenge and superior antitumor activity compared to non-armored anti-NKGD2 CAR-T cells in a murine model [23].

### Ewing sarcoma

Ewing sarcoma (ES) is the second most common primary bone malignancy in the pediatric population after OS. The cornerstone of treatment of ES is chemotherapy induction followed by radiation, surgery, or both. While the 5-year survival rate of localized ES is 75 to 80%, patients with regional or distant metastases have a poorer 5-year survival rate of around 30% [57]. Similar to OS, the heterogeneity of tumor-associated antigens in ES has been a major focus of CAR-T cell development against this disease.

Like neuroblastoma, ES belongs to a group of tumors known as primitive neuroectodermal tumors (PNETs). Since other PNETs had previously demonstrated aberrant expression of GD2, Kailayangiri et al. investigated its expression in ES and demonstrated GD2 expression in 100% of ES cell lines and cell cultures [58]. They engineered GD2-specific CAR-T cells and found that anti-GD2-CAR-T cells lysed ES cells both *in vitro* and in OS xenografts [58].

Charan et al. posited that combining anti-GD2 CAR-T cells with immunotherapy could increase their ability to target and kill ES cells [59]. Hepatocyte growth factor (HGF) appears to be crucial in the formation of a hostile TME by facilitating cross-talk between malignant cells and adjacent stroma [60]. Charan et al. determined that HGF expression was upregulated in ES samples via RT-qPCR and then administered both anti-GD2 CAR-T cells and anti-GD2 CAR-T cells with an adjuvant antibody directed at HGF (AMG102). Superior tumor regression was demonstrated with the anti-GD2 CAR-T cells plus AMG102 compared to the control arm, which did not effectively control the growth of orthotopically implanted primary and metastatic ES [59].

Increased angiogenesis is a hallmark of tumor growth and allows for unchecked vascular proliferation and recruitment of nutrients necessary for tumor survival. The expression of vascular endothelial growth factor receptor 2 (VEGFR2) on ES cells and tumor-associated endothelial cells has been implicated in ES proliferation and correlates with a poor prognosis [61–63]. Englisch et al. investigated the efficacy of VEGFR2-directed CAR-T cells and found that they specifically lysed VEGFR-expressing ES cells in a murine model [64].

Overexpression of the ephrin (EPH) family of receptors is also believed to play a role in the development and progression of several types of cancer, including ES. Funasaka et al. are currently enrolling patients for the CARTiEr study, a single-center, single-arm, phase I study of anti-EPHB4 CAR-T in patients with ES or other EPHB4-positive tumors [65]. Hsu et al. tested EphA2-directed CAR T cells *in vitro* and *in vivo* based on this target’s high expression in ES and OS and low expression in normal bone [66, 67]. These CAR-T cells displayed potent antitumor activity *in vitro* and eliminated ES and OS tumors in a murine model [68]. Additionally, they led to the eradication of OS which had metastasized to murine lungs and livers [68].

Most studies to date have focused on engineering antibodies and CAR-T cells against tumor surface antigens. There have been early successes; however, utilizing proteins secreted by tumors as targets of CAR-T cells. One such cancer-secreted protein is oncofetal tenascin C (TNC). Expression of aberrant isoforms of TNC has been found in several adult tumors [69, 70]. Wickman et al. investigated whether TNC could serve as a target for CAR-T cells in pediatric cancers and found that it was expressed in diffuse intrinsic pontine glioma (DIPG), OS, rhabdomyosarcoma (RMS), and ES [24]. They generated CAR-T cells directed at TNC, which lysed TNC-positive tumor cells *in vitro* but had poor efficacy *in vivo*. To combat the hostile TME, they armored their anti-TNC CAR-T construct with a constitutively active IL-18 receptor (IL-18R). IL-18 had previously been demonstrated to enhance the antitumor activity of both CAR-T cells and endogenous lymphocytes [71]. Accordingly, the anti-TNC CAR-T cells expressing a constitutively active IL-18R displayed superior antitumor effector function *in vitro* and prolonged mouse survival in a murine OS xenograft compared to non-armored anti-TNC CAR-T cells [24].

The G-protein coupled receptor 64 (GPR64) is physiologically expressed only in epididymal tissue; however, it has been noted to be specifically expressed in ES and some other sarcomas [72]. In a conference abstract, Schirmer et al. reported the development of GPR64-directed CAR-T cells which controlled tumor growth in mice bearing ES xenografts [73].

### NB

Neuroblastoma (NB) accounts for 8–10% of pediatric cancers and is the most common extracranial solid tumor in children [74, 75]. Outcomes in NB patients have improved over the past three decades, with a 5-year survival increasing from 52 to 74% [75]. Similar to other solid tumors, the prognosis remains poor for patients with high-risk metastatic disease. Treatment of NB is stratified based on risk, ranging from monitoring or surgery for low-risk cases to high-dose chemotherapy, resection, and stem cell transplantation for high-risk patients with metastatic disease [76]. Treatment of metastatic NB remains one of the primary challenges in the management of this disease, with a 2-year survival rate of approximately 46% [77].

Like pediatric sarcomas, GD2 is highly expressed in most NB cell lines and is, accordingly, a promising target for immunotherapy. It was the first cancer antigen targeted using CAR-T cell therapy for NB due to the previous success of denutuximab, an anti-GD2 monoclonal antibody. The second anti-GD2 mAb for NB, naxitamab, improved on but did not eliminate the side effects of denutuximab, most notably peripheral nerve damage [78].

Early studies involving GD2-directed CAR-T cells demonstrated adequate safety but poor clinical response and CAR-T cell persistence. Pule et al. generated GD2-directed CAR-T cells which persisted *in vivo* for greater than 6 weeks [79]. In a phase 1 clinical trial from 2004 to 2009, children with NB were treated with first-generation CAR-T cells targeting GD2 (NCT00085930). In the first set of results from this trial, Louis et al. reported improved persistence of GD2-directed CAR-T cells up to 192 weeks and demonstrated that even low levels of circulating CAR-T cells were associated with a sustained antitumor response [80]. At an 18-year update in 2025, three of 11 patients with active disease achieved a complete antitumor response, which was sustained in two patients. Of the eight patients with a history of NB but no active disease at the time of infusion, five were disease free at their last follow-up. The overall survival rate was 31.6% at 15 years [81].

Che-Hsing Li et al. reported the long-term outcomes of a clinical trial that ran from 2004 to 2009 of first-generation CAR-T cells targeting GD2 in children with neuroblastoma (NCT00085930). Three of 11 patients with active disease at infusion achieved a complete response. The response was sustained in two patients, one for 8 years and one for more than 18 [81]. Of the eight patients with no disease at the time of CAR-T infusion, five remain disease-free 10 to 15 years after follow-up. Despite these CAR-T cells being first-generation constructs without co-stimulatory domains, they were able to control relapsed and refractory NB in several patients.

A phase 1 and 2 clinical trial of GD2-directed CAR-T cells in pediatric patients with NB or other GD2-positive tumors began in 2018 at the Bambino Gesù Hospital and Research Institute in Rome and remains active (NCT03373097). Del Bufalo et al. reported the first results from this trial in 2023. Twenty-seven children with relapsed or refractory NB received GD2-directecd CAR-T cells. These cells proliferated and remained detectable in blood samples in 26 of the 27 children enrolled up to 30 months after the initial infusion [82]. Seventeen of the 27 children had a positive response, with 9 achieving a complete response and an overall 3-year survival of 60%. Cytokine release syndrome (CRS) occurred in 74% of patients but was mild in all but one. In an updated report of this trial, the total number of children treated with these GD2-CAR-T cells had increased to 54. The overall response rate was 66%, with a complete remission rate of 40% at 6 months and 5-year overall survival rate of 42.7% [83]. CAR-T cells were detected in samples after 12 months in 64% of patients. Four children treated experienced neurotoxicity which was effectively mitigated with the activation of a caspase-9 suicide gene in the CAR-T cells induced by treatment with rimiducid [83]. A follow-up investigation of these results determined that polymorphonuclear myeloid-derived suppressor cells (PMN-MDSCs) impaired the efficacy of the GD2-CARTs in these patients [84]. These cells were found to increase in peripheral blood after treatment with GD2-CAR-T cells and downregulate genes involved in cell activation, inflammatory response, and cytokine secretion. The levels of circulating PMN-MDSCs correlated inversely with the levels of GD2-CAR-T cells in these patients, suggesting that a strategy to limit PMN-MDSC expansion could improve overall CAR-T proliferation [84].

Quintarelli et al. presented a case series of five children with refractory NB who were treated with GD2-CAR-T cells. After treatment, two complete responses were achieved and one was maintained [85]. In addition, one patient had a partial response and one achieved disease stability [85].

Heczey et al. developed a third-generation GD2-directed CAR-T cell after observing poor expansion and long-term persistence with a first-generation construct [86]. They administered these third-generation CAR-T cells in three cohorts: one with the CAR-T cells alone, one with CAR-T cells plus lymphodepletion with cyclophosphamide and fludarabine (Cy/Flu), and one with CAR-T cells, lymphodepletion, and a programmed death-1 (PD-1) inhibitor (NCT01822652). They determined that lymphodepletion prior to CAR-T infusion improved expansion but that the addition of PDL-1 inhibition did not provide an additional benefit [86].

In an abstract, Straathof et al. reported the results of a phase 1 clinical trial in which children with relapsed or refractory NB were treated with escalating doses of GD2-CAR-T cells and lymphodepletion (NCT02761915). No patients had objective clinical response 28 days after infusion; however, six patients receiving a higher dose of CAR-T cells experienced grade 2 to 3 CRS [87]. Three patients did demonstrate regression of soft tissue and bone marrow disease, suggesting that targeting GD2 with CAR-T cells in patients with NB is safe but may require modification to improve CAR-T cell persistence [87].

A GD2-directed CAR-T cell was constructed by Lihua Yu et al. and tested in children with refractory or recurrent NB (NCT02765243). Ten patients were included, with six experiencing stable disease (SD) at 6 months and four with SD at 1 year [77]. Four patients remained alive three to 4 years after infusion. The median overall survival time was 25 months, and the median progression-free survival time was 8 months [77].

Based on the promising results for GD2-directed CAR-T cells in pediatric patients with NB, several fourth-generation armored CAR-T cells have been tested, with more currently in development. Chen et al. investigated the benefits of armoring GD2-CAR-T cells with IL-15 to improve persistence and antitumor function. They constructed both GD2-CAR-T cells and GD2-CAR-T cells with IL-15 incorporated into the CAR cassette and investigated their efficacies *in vitro* and *in vivo* in a murine model. The IL-15-armored CAR-T cells demonstrated superior antitumor activity and survival compared to the non-armored GD2-CAR-T cells. Additionally, they observed that the IL-15 secreted by the GD2-CAR-T constructed produced cells with “memory” and “stem cell-like” phenotypes and was independent of antigen encounter [12].

Fischer et al. constructed GD2-directed CAR-T cells with antigen-inducible IL-18 secretion, which has a favorable side effect profile and induces less systemic toxicity compared to cytokines like IL-12. When tested *in vitro*, the GD2-IL-18-CAR T cells displayed significantly higher quantities of IFN-γ release compared to control CAR-T cells [13]. Accordingly, the cytotoxic effects of these GD2-IL-18-CAR T cells against GD2-positive NB cells were enhanced compared to non-armored GD2-CAR-T cells. Mice treated with the armored variant survived until the end of the observation period of 24 days, when they began to show signs of graft-versus-host-disease (GVHD). Armored anti-GD2 CAR-T cells expressing IL-18 were also manufactured by Glienke et al. and co-cultured with GD2-negative and GD2-positive NB cell lines. They demonstrated high levels of cytokine release and cytolytic factors compared to background cytokine levels and efficacious killing of GD2-positive NB cells [14].

Li et al. constructed GD2-directed CAR-T cells which expressed both IL-7, a cytokine with promising antitumor effects, and CCR2b, a chemokine receptor which migrates towards the cancer chemokine CCL2. In a mouse NB model, only GD2-CAR-T cells co-expressing IL-7 and CCR2b inhibited tumor growth, unlike classical CAR-T cells and mock CAR-T cells [15]. These studies suggest that armoring GD2-directed CAR-T cells in NB can produce a potent antitumor effect, and that utilization of interleukins with more favorable side effect profiles can reduce systemic toxicity.

When GD2-directed CAR-T cells were fitted with p40, a subunit of IL-23, Ma et al. observed that these cells produced IL-23 upon T-cell stimulation [16]. In a metastatic NB model in mice, these cells exhibited superior antitumor activity without side effects compared to CAR-T cells engineered with IL-18 or IL-15, which produced weight loss in the mice. They hypothesized that p40 CAR-T cells were able to achieve superior antitumor effects by selectively secreting IL-23 without high levels of production of other cytokines, which would lead to more deleterious inflammatory effects [16].

Heczey et al. reported the interim results from the GINAKIT2 trial of GD2-specific CAR-NKT cells secreting IL-15 in children with neuroblastoma. CAR-NKT cells utilize a CAR similar to conventional CAR-T cells but present several advantages, including native expression of constitutively active chemokine receptors and superior activation of surrounding NK and T cell responses [26]. No dose-limiting toxicities occurred among the 12 patients enrolled [26]. The objective response rate was 25%, with one complete response and two partial responses [26].

B7-H3 is also highly expressed in NB cell lines [88]. Du et al. studied B7-H3-CAR-Ts to treat three types of cancer: neuroblastoma, ovarian cancer, and pancreatic ductal adenocarcinoma. They studied their efficacy *in vitro* and in an orthotopic murine model. B7-H3-directed CAR-T cells controlled the growth of the tumors in both environments [89]. Birley demonstrated similar efficacy of B7-H3-directed CAR-T cells in a resistant murine NB model [90]. Moghimi et al. armed anti-B7-H3 CAR-T cells with a TGF-β switch receptor (CSTR) which converted the normally immunosuppressive signal into a TLR-mimicking response. They observed that CSTR reversed the inhibitory effect of TGF-β on these CAR-T cells, which had superior and persistent antitumor efficacy compared to B7-H3 CAR-T cells alone [20]. In orthotopic models of NB, ES, and OS, these CAR-T cells were effective at penetrating tumors and controlling disease, while unmodified B7-H3 CAR-Ts failed [20].

Glypican 2 (GPC2) is an oncofetal antigen highly expressed in NB and other pediatric cancers [91]. Okada et al. manufactured anti-GPC2 CAR-T cells armored with IL-15 and IL-21 and evaluated their efficacy *in vitro* and *in vivo* in a mouse model. They tested three constructs: anti-GPC2 CAR-T cells with constitutively secreted IL-15 and IL-21, membrane-bound IL-15 and IL-21, and NFAT-inducible membrane-tethered expression of both cytokines [25]. All three versions enhanced NB cell killing relative to control anti-GPC2 CAR-T cells.

Another novel immunotherapeutic technique involves recruiting nearby endogenous immune cells to assist in the antitumor response. Pascual-Pasto et al. tested GPC2-directed CAR-T cells which secreted a bispecific innate immune cell engager (BiCE) targeting both GD2 and CD16a, a marker expressed by innate immune cells in the TME [92]. *In vitro*, these CAR-T cells promoted GPC2-dependent tumor cell killing and secreted GD2.BiCE, which promoted activation of antitumor innate immune cells. *In vivo*, the CAR-T constructs successfully delivered the GD2.BiCE, which improved intratumor retention of native NK cells and enhanced CAR-T cell efficacy [93].

The search for other antigen targets for NB continues. Receptor tyrosine kinase-like orphan receptor 2 (ROR2) is overexpressed in NB and promotes cell growth and metastasis. CAR-T constructs targeting ROR2 showed specific cytotoxicity towards ROR2-positive cell lines *in vitro*, with *in vivo* efficacy currently being studied [94]. Roundabout guidance receptor 1 (ROBO1) encodes a protein receptor used in cell migration and was shown to be highly expressed in NB and RMS using cell surface proteomics [95]. Anti-ROBO1 CAR-T cells eliminated tumor cells *in vitro* and led to tumor regression in ROBO1-positive tumor-bearing mice [95]. Additionally, CAR-T cells directed at anaplastic lymphoma kinase (ALK), a potent oncogenic driver in NB, were an effective form of monotherapy in NB samples with high ALK expression [96]. A phase I clinical trial is currently underway to determine the maximum tolerated dose of ALK-directed CAR-T cells in children with relapsed or refractory high-risk NB (NCT06803875). NKG2D, an activating receptor on NK cells, has proven to be a successful antigen target in OS and is being studied by Clares-Villa et al. in a preclinical study for NB. At an abstract at the International Conference of Lymphocyte Engineering in 2023, they announced they are developing an NKG2D-directed armored CAR-T cell which releases IL-18 upon activation [97].

### DIPG/DMG

Since their initial characterization in 1926, diffuse intrinsic pontine glioma (DIPG) and diffuse midline glioma (DMG) have remained universally fatal brainstem tumors affecting children. Radiation remains the standard of care, but it is not curative and is associated with recurrence [98]. Because it is surgically unresectable and carries a high mortality rate, immunotherapy has been investigated as a possible treatment option for DIPG. As in other pediatric solid tumors, GD2, B7-H3, and HER2 are also highly expressed in DIPG [98].

After establishing high GD2 expression on H3K27M-mutated glioma cells and testing GD2-directed CAR-T cells in a preclinical setting, Majzner et al. launched a first-in-human phase I clinical trial of GD2 CAR-T cells in children with DIPG or DMG (NCT04196413). Three of the four patients enrolled showed clinical and radiographic improvement without on-target, off-tumor toxicity [99]. In an updated report of this trial, thirteen patients had been enrolled and 11 had received infusions of the GD2-CAR-T cells, 9 with DIPG and 2 with DMG. After performing a dose escalation, four patients demonstrated major reductions in tumor volume, and three exhibited small reductions [100]. Nine patients have shown clinical neurological improvement, and one patient has demonstrated a complete response for over 30 months since they were enrolled in the trial [100].

To improve the effectiveness of GD2-directed CAR-T cells in DMG, De Billy et al. co-administered these cells with linsitinib, an IGF-1R inhibitor and noted enhanced antitumor activity compared to GD2-CAR-T cells administered without linsitinib both *in vitro* and *in vivo* in an orthotopic murine xenograft model [101]. Lin et al. fitted a GD2-directed CAR-T cell with a constitutively active IL-7 receptor and treated patients with GD2-expressing DMG who received pretreatment radiation or chemotherapy (NCT04099797). Patients receiving these CAR-T cells showed a temporary improvement in neurologic deficits, and seven of eight were eligible for additional treatment cycles [27].

B7-H3 is the most extensively investigated antigen target for pediatric patients with DIPG and DMG. After launching the Brainchild-01 and Brainchild-02 trials of HER2- and EGFR-specific CAR-T cells in pediatric CNS tumors, Seattle Children’s Hospital began work on Brainchild-03, a phase I clinical trial of B7-H3 CAR-T cells for children with DIPG/DMG or recurrent or refractory CNS tumors (NCT04185038). In 2025, Vitanza et al. reported the results from Arm C of the trial, which was restricted to patients with DIPG. Twenty-one patients were treated with repeated doses of B7-H3 CAR-T cells, which conferred a median survival from initiation of 10.7 months and median survival from diagnosis of 19.8 months, superior to the median overall survival of DIPG of 11 months from diagnosis [102]. Three patients remained alive at the time of publication [102].

Clinical benefits in the Brainchild-03 study were not uniformly seen, so Timpanaro et al. sought to utilize multi-modality therapy to improve responses. They tested the same B7-H3 CAR-T cells in a preclinical setting and added ONC206 to the regimen, an imipridone molecule that can induce anti-proliferative effects on cancer cells [103]. The addition of ONC206 enhanced CAR-T cytotoxicity *in vivo* and extended the survival of mice with DIPG xenografts [103].

Myers et al. postulated that part of the reason behind the non-uniform responses to their previous studies was heterogeneity of tumor antigens. They generated quad-CAR-T cells targeting HER2, EGFR806, B7-H3, and IL-13ra2 and conducted preclinical testing using them. *In vitro*, they demonstrated significant killing of cancer cells and cytokine production compared to mock CAR-Ts [104]. A xenograft mouse model of DMG showed a significant survival advantage when treated with these quad-CAR-T cells [104]. Based on this preclinical testing, they began the Brainchild-04 clinical trial of quad-targeting CAR-T cells in pediatric CNS tumors (NCT05768880). In an abstract, Ronsley et al. the first set of results from this trial, which has enrolled six patients with DMG, five with DIPG, and four with other CNS tumors [105]. No dose-limiting toxicities, cytokine release syndrome, or immune effector cell-associated neurotoxicity syndrome have been experienced, and all patients remain alive with a median follow-up time of 93 days [105].

To improve the trafficking of CAR-T cells towards target tumors, Song et al. engineered B7-H3 CAR-T cells to overexpress the chemokine receptor CXCR3 [21]. In their recent clinical trials, they had observed locoregional elevation of CXCR3’s ligand, CXCL10. When tested *in vitro* in a trans-well chemotaxis assay, CXCR3-modified B7-H3 CAR-T cells effectively migrated towards CXCL10 in a dose-dependent manner [21]. In an orthotopic mouse model of DIPG, CAR-T cells modified with CXCR3 maintained complete tumor regression, whereas four of five mice treated with unmodified B7-H3 CAR-T cells experienced rebound of tumor signals [21].

After establishing high levels of HER2 expression on patient-derived DIPG cells, Wang et al. constructed second-generation HER2-CAR-T cells. These cells exhibited potent and specific cytotoxicity both *in vitro* when co-cultured with DIPG cells and *in vivo* in a DIPG xenograft model [106].

In a 2022 abstract, Ferrerosa et al. described creating CAR-T cells expressing interleukin-13 receptor α2 (IL-13Rα2) and ephrin type A receptor (EphA2). Three of five mice with DIPG who were given these CAR-T cells demonstrated complete remission, although the tumors did not completely regress [107].

In concordance with previous results, Foster et al. found high GPC2 expression among pediatric CNS malignancies, including medulloblastomas and embryonal tumors [108]. GPC2-directed CAR-T cells prolonged survival in an orthotopic mouse model of DMG, with no systemic toxicity observed [108].

### Rhabdomyosarcoma

Rhabdomyosarcoma (RMS) is an aggressive malignant neoplasm that arises from striated muscle and primarily affects children. The prognosis of RMS is poor, with a 30% 5-year survival rate for RMS patients with metastatic disease or patients with moderate to high-risk RMS [109].

Several tumor antigens overexpressed in RMS have been studied as potential CAR-T targets. Lavoie et al. found B7-H3 to be overexpressed by most RMS tissue samples and noted an inverse variation between high B7-H3 expression and lower surrounding CD8 + T cell density, suggesting that B7-H3 may play a role in immune evasion [110]. Piccand et al. created a CAR-T cell co-expressing receptors for L1CAM and B7-H3, which they confirmed were both highly expressed in RMS [111]. These cells persisted for up to 50 days post-injection and were highly potent against RMS cells which expressed low levels of L1CAM, indicating the potential for logic-gated CAR-T cells [111].

Fibroblast growth factor receptor 4 (FGFR4), which plays crucial roles in cell proliferation and differentiation, is perhaps the most promising target antigen for CAR-T therapy in RMS. Khan et al. performed immunohistochemistry assays on samples of small round blue cell tumors (SRBCTs) and found moderate to strong FGFR4 staining in all 26 RMS samples tested, including both alveolar and embryonal subtypes [112]. In a subsequent study, Xiao et al. engineered an FGFR4-directed CAR-T cell with an inducible caspase-9 suicide gene system for increased safety [113]. The *in vitro* and *in vivo* tumoricidal effects of FGFR4 CAR-T cells were significant, leading to reduced tumor volume in a mouse model and no luminescence signal in the subcutaneous tumor area in the FGFR4 treatment group [114]. Their toxicity was low, as indicators of mouse health in this group were optimal. This study was limited by utilizing a subcutaneous model of RMS rather than an orthotopically implanted one, which limits the ability to replicate a native tumor and its surrounding TME. Cheuk et al. successfully generated binder molecules specific to FGFR4, and CAR-T cells developed from these binders demonstrated cytotoxicity against FGFR4-positive RMS cells in a murine model [115]. In a preclinical study by Alijaj et al., FGFR4-CAR-T cells displayed potent and specific cytotoxicity to RMS cells expressing FGFR4 [116].

Sullivan et al. conducted an *in vitro* preclinical study of FGFR4-binding moieties to be used as CARs. After confirming cytokine production and cytotoxicity in the presence of RMS cell lines, they selected two to move forward for *in vivo* murine testing [117]. They then tested FGFR4-directed CAR-T cells in a murine model of orthotopic RMS. Due to a limited initial response to treatment, they investigated the potential interfering effects of the TME and found high expression of macrophage migration inhibitory factor (MIF), transforming growth factor β1 (TGF-β1), and leukemia inhibitory factor (LIF) [118]. These soluble factors have generally immunosuppressive effects and portend a poor prognosis in several cancers [118]. To augment their CAR-T cell treatment, they co-administered pharmaceuticals to inhibit these factors and noted extended survival of the mice compared to FGFR4 CAR-T cells alone [118].

Due to the high expression of both antigens on RMS samples, Tian et al. created bicistronic CAR-T cells targeting both FGFR4 and B7-H3. Whereas CAR-T cells targeting FGFR4 alone demonstrated limited cytotoxicity in their experiments, the bicistronic CAR-T cells displayed more potent killing of RMS cells *in vitro* and faster cancer eradication, increased persistence, and reduced exhaustion marker expression in an orthotopic murine RMS model [119]. Timpanaro et al. also engineered CAR-T cells targeting both FGFR4 and B7H3 by optimizing the earlier model created by Alijaj et al. These dual-targeted CAR-T cells facilitated clearance of orthotopic RMS in five of five mice tested [120].

HER2 has also been found to be overexpressed on samples of RMS in adults and children [121]. HER2 was expressed in six out of six RMS cell lines studied by Lulu et al. [122]. Anti-HER2 CAR-T cells and anti-α_v_β_3_ (avb3, an integrin postulated to be involved in cancer angiogenesis) both robustly killed RMS in a dose-dependent manner [122]. Importantly, a separate study by Lulu et al. demonstrated the ability of surviving tumor cells to generate a suppressive TME. After anti-avb3 and anti-HER2 CAR-T administration, surviving tumor cells upregulated expression of exhaustion ligands and circulating CAR-T cells showed increased expression of exhaustion markers [123].

In 2024, Hegde et al. presented updated results of the clinical trial of HER2 CAR-T cells in advanced sarcomas (NCT00902044). Four patients with metastatic RMS, eight patients with OS, and one patient each with a PNET and synovial sarcoma had been treated with HER2 CAR-T cells and lymphodepletion. Fifty percent of patients had experienced disease stabilization or remission [124]. One 7-year-old patient with metastatic RMS enrolled in this trial has achieved durable remission 20 months after his third infusion of HER2 CAR-T cells [125].

In a clinical case report, Jiang et al. treated a 2-year-old male patient with RMS with anti-CD56 CAR-T cells whose disease had persisted despite surgical and medical interventions. More than 3 years after the intervention, the patient was in complete remission and had demonstrated no evidence of CRS [126].

In the aforementioned study by Pezzella et al., GD2-directed CAR-T cells were also tested in murine models of disseminated RMS [32]. Mice treated with GD2-CAR-T cells survived an average of 69.5 days after infusion, significantly longer than RMS mice treated with control CAR-T cells [32].

Wei Xiao et al. developed a CAR-T cell that targets platelet-derived growth factor receptor alpha (PDGFRA), which they determined to be expressed at higher levels in RMS compared to normal human and mouse skeletal muscles [113]. PDGFRA CAR-T cells significantly inhibited the growth of RMS in a murine xenograft model.

Shraim et al. determined that the cell surface protein GPC2 was overexpressed in 16 of 21 RMS models studied [127]. Compared to control CAR-T cells, their GPC2-CAR-T cells displayed potent toxicity and specificity against RMS cells in a murine RMS xenograft model [127].

### Retinoblastoma

Retinoblastoma (RB), caused by a loss-of-function mutation in the Rb1 tumor suppressor gene, is the most common ocular malignancy of childhood [128]. Its survival rate is relatively high compared to other pediatric tumors, as enucleation of the affected eye is largely curative. However, children with inherited RB due to a germline mutation in Rb1 most often have bilateral disease, and a minority of patients experience intracranial tumors [129]. Due to the poor prognosis of metastatic disease and a focus on eye-preserving treatments, immunotherapy is being investigated for therapeutic benefit in RB.

CAR-T cells have shown early promise in treatment of RB both *in vitro* and *in vivo.* Like in other neuroectodermal tumors, GD2 is highly expressed in RB. CD171, or L1CAM, is a cell adhesion molecule found in CNS cells that was also recently shown to be highly expressed in RB [130]. Andersch et al. designed CAR-T cells directed against CD171 and GD2 and assessed their efficacy at killing RB cells in vitro [130]. CD171- and GD2-directed CAR-T cells demonstrated significantly improved killing of RB cells compared to control CAR-T cells. It was noted, however, that the mice treated with CD171-directed CAR-T cells showed a transient loss of antigen expression in the target cells which was not observed for cells treated with GD2-directed CAR-T cells, suggesting that antigen downregulation may pose a challenge in certain solid tumors. Sujjitjoon et al. also studied the use of GD2-CAR T cells against RB and noted effective killing of tumor cells. This study did note GD2 downregulation in tumor cells, which was partly attributed to the inhibitory PD1–PD-L1 interaction between CAR-T cells and RB cells [131].

Pascual-Pasto et al. determined that the oncofetal antigen GPC2 was expressed on RB tumors but not healthy retinal tissue, which is consistent with findings among other pediatric malignancies [92]. In murine models of RB, both intraventricularly and systemically administered GPC2-directed CAR-T cells with a 41BB co-stimulatory domain significantly extended mouse survival compared to control CAR-T cells [93].

Wang et al. also used GD2 as a CAR-T target in RB but encapsulated it in an injectable hydrogel and armored it with IL-15. This construct was studied in a population of mice with xenografted RB tumors in the subretinal space to simulate the anatomical presentation of RB. In the group of mice treated with the IL-15-releasing CAR-T cells, there was significant antitumor activity, with 60% of these mice remaining tumor free up to day 70 [17]. The antitumor activity of the GD2-CAR-T cells was only noted upon inclusion of the IL-15 construct and delivery with an injectable hydrogel, indicating that armoring with cytokines and inclusion of a delivery vessel improved their cytotoxicity against RB.

### Wilms tumor

Wilms tumor (WT), which originates from persistent metanephric tissue, is the fourth most common pediatric cancer. It carries a favorable prognosis, with a 5-year survival rate of 92% in the USA [132]. The standard of treatment is nephrectomy, which is typically curative. Bilateral disease, however, presents a challenge as bilateral nephrectomy necessitates lifelong dialysis. Immunotherapeutic modalities, such as CAR-T cells, may represent less invasive treatment options for children affected by WT.

Crucial to the development of CAR-T cell therapy for WT patients is the identification of appropriate target antigens. B7-H3, EGFR, and GPC3 have all been confirmed to be highly expressed in samples of WT [48, 133, 134]. Seidmann et al. identified another oncofetal membrane protein, Claudin 6 (CLDN6), as one such target. They reported expression of CLDN6 in 40% of WT samples and low expression in normal tissue [135].

Compared to other pediatric cancers, the number of CAR-T cells tested specifically in WT remains low. There are, however, several clinical trials for CAR-T cells which target antigens highly expressed in samples of WT. The STRIvE-01 trial of EGFR-806-directed CAR-T cells is open to children with EGFR-positive tumors, including WT. EGFR has been found to be expressed in a number of pediatric tumors, including WT, and is postulated to play a role in its development [136]. In an abstract, Albert et al. reported acceptable safety profiles of these CAR-T cells among the eleven patients that have been enrolled [133].

Steffin et al. reported the results of several clinical trials in children and adults of GPC3-directed CAR-T cells, an oncofetal antigen highly expressed in WT (NCT05103631 and NCT04377932) [28]. One cohort received GPC3 CAR-T cells alone, while another cohort received GPC3 CAR-T cells armored with IL-15, which significantly increased CAR-T expansion and induced superior disease control rates compared to the non-armored CAR-T cells [28]. Of the cohort which received the IL-15 armored CAR-T cells, one had WT, one had RMS, and the remainder had HCC or other unspecified hepatocellular cancers [28]. The patient with WT experienced progressive disease, while the patient with RMS exhibited a partial response to treatment [28]. 

## Future directions

### Minimizing cytotoxicity

**Fig. 2:**
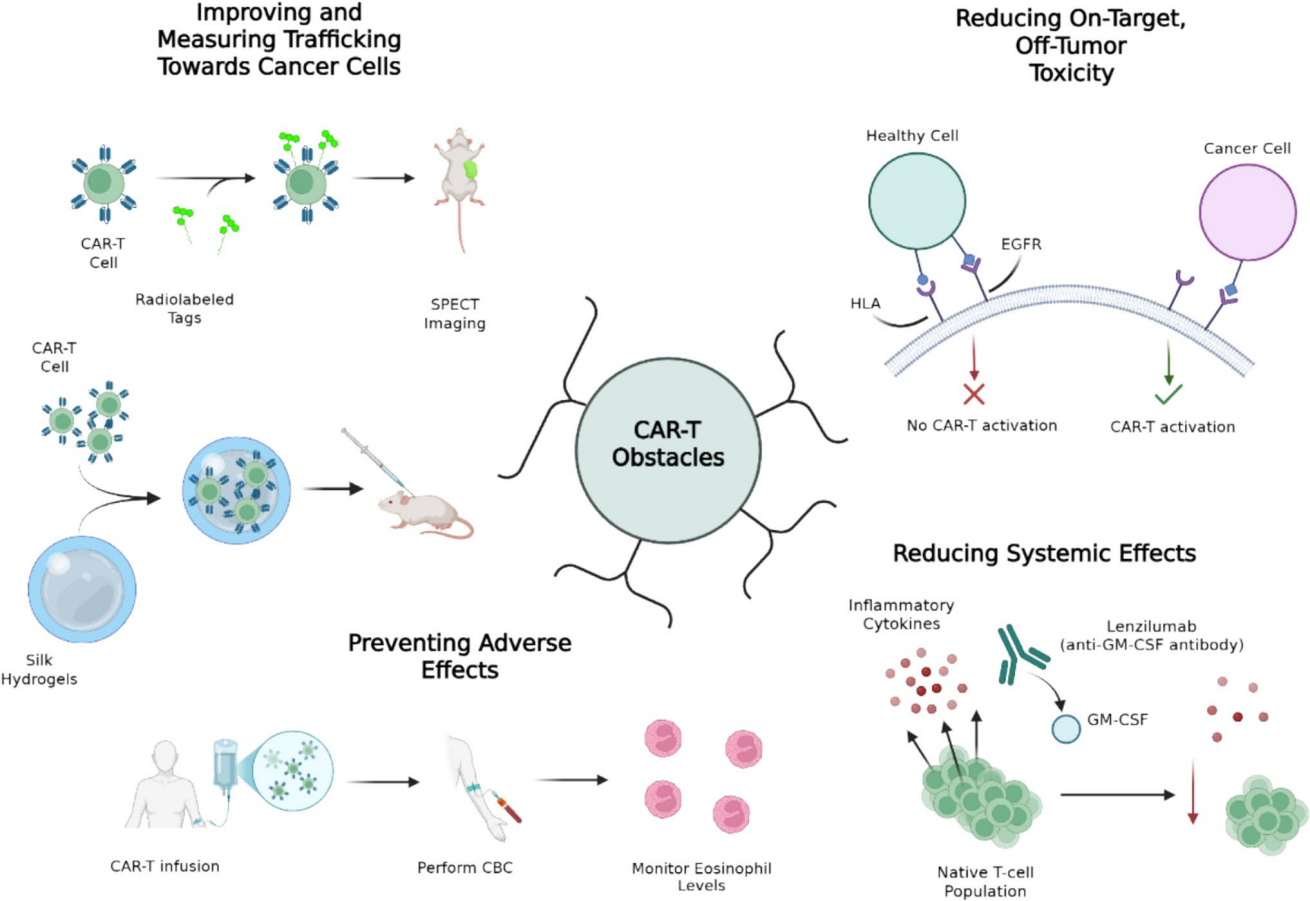
Current obstacles in CAR-T cell treatments in pediatric solid tumors and advancements in development to address them. Bicistronic logic-gated CAR-T cells circumvent on-target, off-tumor toxicity by ensuring that multiple tumor antigens are recognized before exerting cytotoxic effects. Administering CAR-T cells using silk hydrogels and measuring their location using fluorescent markers shows promise in improving trafficking. Co-administration of anti-GM-CSF mAbs was demonstrated to dampen deleterious systemic inflammation in CAR-T patients. Monitoring peripheral blood markers, such as eosinophilia, may indicate which patients are likely to develop adverse effects to treatment. Created in BioRender. Genetics, C. (2026) https://BioRender.com/bfcrbbv

Several obstacles still hinder CAR-T cell efficacy in solid tumors and remain active areas of research (Fig. 2). CAR-T cells, especially armored variants which release inflammatory cytokines, have been associated with significant systemic toxicities, such as CRS and neurotoxicity. Early studies of CD19-directed CAR-T cells showed antitumor efficacy, but up to 50% of patients treated with these cells develop grade 3 or higher CRS or neurotoxicity [137–140]. These studies also found that the pathogenesis of CRS was related to the expansion of T cells *in vivo* and the production of effector cytokines by T-cells, such as GM-CSF. The administration of anti-CD19 CAR-T cells with concomitant Lenzilumab, an anti-GM-CSF monoclonal antibody, led to a reduction of neurotoxicity and CRS with preserved antitumor efficacy in a murine model [33]. This suggests that the use of monoclonal antibodies directed at inflammatory cytokines may mitigate systemic toxicities associated with CAR-T cell therapy.

Improving the on-target activity of CAR-T cells and mitigating off-target and off-tumor activity is another avenue for ameliorating systemic toxicity. On-target, off-tumor toxicity refers to CAR-T cells which engage the target antigen but on healthy cells rather than tumor cells. Off-target toxicity, conversely, occurs when CAR-T cells engage with antigens other than the target antigen. Kirtane et al. found that monoclonal antibodies that target EGFR, which is implicated in tumorigenesis, demonstrate impressive efficacy but are limited by off-tumor toxicity such as skin rashes [141]. To overcome this, they developed A2B385, a logic-gated CAR-T cell that targets EGFR. It included 2 CAR domains: an activator which recognizes EGFR on both tumor and normal cells and a blocker portion which targets HLA-A02 and inhibits CAR-T activity against normal cells via loss of HLA expression on tumor cells. Activation of this CAR-T construct requires stimulation of the EGFR-recognizing activator domain and non-activation of the HLA-A02 targeting blocker domains, decreasing the risk for graft-versus-host disease. This construct is currently being used in the DENALI-1 trial, a phase 1/2 open-label study evaluating the safety and efficacy of A2B395 in adults. EVEREST-2, a trial of the logic-gated CAR-T cell A2B694 in patients with solid tumors associated with mesothelin expression, is also underway. Of the five participants enrolled so far, no dose-limiting toxicities, including CRS or neurotoxicity, have been noted [142].

Such logic-gated CAR-T cells are also being studied for use in DIPG, as Venkataraman et al. tested a novel bicistronic CAR that requires concomitant activation of both B7-H3 and CD99 due to their uniquely high levels of expression in DIPG. These constructs were able to effectively kill tumor cells and demonstrated no cytotoxicity against cells expressing either antigen alone. They completely cleared DIPG cells in murine models and led to increased survival compared to monovalent CAR-T cells [143]. Incorporating logic-gated domains such as this in armored CAR-T cells may also reduce their off-target toxicity and could be expanded to pediatric solid tumors.

### Multi-modality therapy

Combining CAR-T cells with other modalities, such as radiotherapy, pharmacotherapy, or genetic therapy, is an attractive option for increasing potency and minimizing off-target toxicity. Soderholm et al. combined GD2-CAR-T cells with ultra-high-dose radiotherapy, which reduced damage to normal brain tissue and preserved control of DIPG. The expression of GD2 on DIPG cells was unchanged after radiotherapy, indicating that it did not significantly alter target antigen expression [144]. The use of external beam radiotherapy (EBRT) with anti-B7-H3 CAR-T cells in mice with NB was also effective in improving survival rates, as mice with primary NB treated with CAR-T cells alone had a median survival of 81 days, but all mice treated with EBRT plus CAR-T cells survived disease-free past 130 days [145]. Ansari et al. demonstrated similar findings in murine models of disseminated metastatic NB [145]. Total body irradiation administered 1 day prior to anti-B7-H3 CAR-T infusion also vastly enhanced the efficacy of this treatment in a study by Barisa et al. [146].

Autophagy is a cellular mechanism that supports tumor survival and regulates immune interactions. De Mitri et al. hypothesized that inhibiting autophagy could increase the efficacy of CAR-T cell therapy in NB and combined anti-GD2 CAR-T cells with an inhibitor of ULK1, a protein kinase crucial to autophagy. *In vitro* assays were significant for increased CAR-T-mediated tumor cell killing, and *in vivo* studies demonstrated prolonged tumor control compared to CAR-T cells administered without an autophagy inhibitor [147].

Grunewald et al. noted that L1CAM-targeting CAR-T cells had a limited response against NB cells and found that NB cells with high MYCN levels expressed lower levels of L1CAM. They administered MLN8237, an MYCN inhibitor, and subsequently demonstrated increased expression of L1CAM on tumor cells and enhanced cytotoxicity of anti-L1CAM CAR-T cells [148].

Using an NB model, Launspach et al. used CRISPR to insert pro-inflammatory cytokines (CXCL10, CXCL11, and IFNG) into tumor cells to reshape the immunosuppressive TME. This increased the ability of CAR-T cells to infiltrate the tumor and increased their cytotoxic efficacy both *in vitro* and *in vivo*, indicating the potential for genetic therapy to ameliorate the TME and prime it for CAR-T cell activation [149]. Rossari et al. used a different approach to reshape the immunosuppressive TME by infusing tumor-associated macrophages which released immunostimulatory IFN-alpha and/or orthogonal IL-2 along with anti-B7H3 CAR-T cells. These additional engineered tumor-associated macrophages were shown to rescue the functionality of CAR-T cells by inhibiting exhaustion and induced effector and memory states [150].

### Tumor heterogeneity

Despite the identification of overexpressed tumor markers, tumor heterogeneity remains a challenge in the treatment of pediatric solid tumors. Targeting multiple tumor antigens with a single CAR-T cell is a potential strategy to overcome this limitation. Timpanaro et al. designed CAR-T cells which targeted both CD276 (B7-H3) and FGFR4 to combine the previously established efficacy of both target antigens [120]. Tian et al. analyzed protein and transcriptome expression of CAR-T cells against NB to identify which CAR-T targets had the highest activity. Using this data, they constructed bicistronic CAR-T cells targeting both GPC2 and CD276. These cells demonstrated improved persistence, cytotoxic activity, and resistance to exhaustion compared to CAR-T cells targeting either antigen alone [151]. The aforementioned study by Myers of quad-targeting CAR-T cells for DMG has also presented encouraging early results.

Wu et al. harnessed the high expression of both GD2 and GPC2 in NB and constructed CAR-T cells which targeted both tumor markers. These bispecific CAR-T cells had binding affinity similar to CAR-T cells specific to either GD2 or GPC2 alone and extended antitumor activity through the release of other proinflammatory cytokines [152]. Strijker et al. found that macrophage migration inhibitory factor (MIF), a component of the TME, inhibited CAR-T cell activation and cytotoxicity in vivo [153]. They explored the ability of PROTAC, a novel class of proteolytic molecules, to target and degrade MIF. They found that MIF-targeting PROTAC increased activity of bicistronic CAR-T cells directed at B7-H3 and GPC2.

### Predicting response to CAR-T cell therapy

Currently, there are no reliable methods to predict which patients will respond to CAR-T cells therapy and which are at the highest risk for developing complications. Trautwein et al. developed Cu-NOTA-ch13.18/CHO, a radiolabeled anti-GD2 antibody that allows for assessment of GD2 positivity via PET before administration of anti-GD2 CAR-T cell therapy to individual patients [154]. Ghosh et al. took a similar approach for the antigen B7-H3 and developed a radiolabeled anti-B7-H3 antibody conjugated with Zr-89 as a PET tracer [155]. The level of GD2 and B7-H3 positivity may serve as a useful screening tool and an indicator for how well these patients will respond to anti-GD2 CAR-T cell therapy, although this remains to be seen. Kaczanowska et al. evaluated the immune profiles of patients with GD2-positive tumors that received CAR-T cells, including NB and OS, to ascertain the characteristics of patients who responded well. Good responders showed increased abundance of naïve CD8 + T cells, and poor expanders demonstrated higher expression of T cell exhaustion markers post-treatment [156]. Chen et al. found that nonresponders to GD2-CAR-T cell treatment for DMG showed elevated signatures of regulatory T cells (Tregs) in the CSF after treatment [157]. These findings suggest that a favorable population of native T cells prior to CAR-T administration could contribute to favorable treatment results.

A study by Fatima et al. found that the absence of peripheral eosinophilia could serve as a marker for an increased probability of developing systemic complications of CAR-T cell treatment [158]. This study examined patients who had received CD-19-directed CAR-T cells for the treatment of B-cell lymphoma and split them into 2 groups of patients: those who had developed eosinophilia on lab testing, and those who had not. After propensity score matching was performed, the group with peripheral eosinophilia had a significantly lower risk of CRS, immune effector cell-associated neurotoxicity syndrome (ICANS), and immune effector cell–associated hematotoxicity (ICAHT) [158]. Infection-free survival was higher in the eosinophilia group, while overall survival was not significant. The use of eosinophilia to stratify CAR-T cell patients by risk may prove useful in identifying those who are at higher risk for CRS, ICANS, and ICAHT. Expanding and validating this method in pediatric patients receiving novel CAR-T cell therapy for solid tumors may help stratify risk in this group as well.

### Trafficking

Improving trafficking of CAR-T cells towards solid tumors remains an important obstacle that, if overcome, could expand the usefulness of next-generation armored CAR-T cells in pediatric solid tumors. Li et al. used injectable silk hydrogels to deliver B7H3-directed CAR-T cells to mice with subcutaneous melanomas [159]. Subcutaneous administration at sites distal and proximal to tumors led to complete tumor clearance. Additionally, the CAR-T cells were detectable in peripheral blood samples 3 weeks after treatment, indicating that the silk hydrogel facilitated retention and viability of the cells. Promising results were also noted in a murine model of bladder cancer utilizing the same hydrogel delivery system and CAR-T construct.

A different study attempted to manipulate the TME by using anti-VEGF antibodies [160]. The overexpression of VEGF in solid tumors leads to aberrant peritumoral vascular development and facilitates the delivery of nutrients to malignant cells. Dong et al. engineered a murine model of glioblastoma which expressed EGFRvIII, one of the most common neoantigens in GBM, and delivered CAR-T cells directed to this antigen. In one mouse group, they concomitantly administered an anti-mouse VEGF antibody. The group treated with the anti-VEGF antibody and anti-EGFRvIII CAR-T cells demonstrated improved CAR-T cell infiltration into the TME, delayed tumor growth, and increased mouse survival duration compared to the group treated with CAR-T cells alone.

Lake et al. created B7-H3 CAR-T cells that expressed CXCR2, an IL-8 receptor, and found that overexpression of this receptor significantly enhanced homing into IL-8 expressing tumors such as OS [18]. Similarly, Talbot et al. identified IL-8 and CXCL16 as the chemokines most highly expressed by OS and modified anti-B7-H3 CAR-T cells with CXCR2 as well as CXCR6, the cognate receptor for CXCL16 [19]. These modifications enhanced CAR-T cell homing towards OS cells both *in vitro* and *in vivo*, including in a metastatic murine OS model.

Parra-Nieto et al. utilized an approach that involved co-administration of CAR-T cells with nanometric bispecific T cell engagers (NBTEs) to improve trafficking towards solid tumors. These NBTEs expressed a receptor for an overexpressed norepinephrine transporter overexpressed on NB cells as well as fluorescein, which was recognized by anti-FITC CAR-T cells [161]. NB cells incubated with NBTEs were rapidly detected and killed by anti-FITC CAR-T cells. This dual approach facilitated tumor monitoring with fluorescence microscopy and allows for tuning of NBTEs towards different target antigens without modification of anti-FITC CAR-T cells, paving the way for their utilization in a number of different malignancies.

Hidalgo et al. took a similar approach for patients with OS and administrated anti-FITC CAR-T cells with an anti-B7-H3 monoclonal antibody conjugated to FITC. The monoclonal antibodies were able to penetrate the tumor and were bound by anti-FITC CAR-T cells which exerted cytolytic effects [40]. A phase I trial for patients with refractory OS is currently underway which involves infusing anti-FITC CAR-T cells along with the high-affinity UBTT170 tumor tag conjugated to fluorescein [162].

Using CAR-T cells which are radiolabeled with small molecules or nanoparticles and can be detected by SPECT or PET is a novel technique which allows for the detection of the cells in solid tumors [163]. To map effective CAR-T trafficking and infiltration into the TME, Plummer et al. utilized a multi-omics assay combining next-generation RNA fluorescence* in situ* hybridization (FISH) with protein co-detection and used it in a pediatric patient who is part of a clinical trial for CAR-T cell therapy in solid tumors [164].

### Lymphodepletion

Lymphodepletion (LD) has become an essential adjuvant treatment administered with CAR-T cells for the treatment of hematologic malignancies, as it reduces the native lymphocyte population and allows for the expansion of transduced CAR-T cells. LD has also become more common in CAR-T cell trials for solid tumors, with a review of clinical results from CAR-T trials for solid tumors published since 2010 noting that 24 out of 31 trials employed LD [165]. In a 2017 study of GD2-directed CAR-T cells for neuroblastoma, a cohort of patients that underwent LD exhibited superior CAR-T expansion and survival duration compared to a cohort without LD [86]. In another study, patients with advanced sarcoma were started on a low dose of CAR-T treatment without LD and gradually increased to higher doses. Even at the highest dose, few patients demonstrated adequate CAR-T levels of 1000 copies per nanogram or higher. When this was repeated with initial LD treatment, there was a 3.5-fold increase in the maximum CAR-T transgene level [36].

## Conclusion

CAR-T cell therapy has become an increasingly common treatment for hematologic cancers in adults and children, but progress in solid tumors has lagged. This is due to factors including tumor antigen heterogeneity, issues with trafficking CAR-T cells to tumor sites, and difficulty penetrating the hostile TME. Armored CAR-T cells have shown tremendous promise in circumventing the hostile TME through the addition of antigen-inducible cytokines or cytokine receptors, which modify the environment to facilitate cytotoxicity against tumor cells. Results for CAR-T cells, especially armored CAR-T cells, in *in vitro* and murine xenograft models have been encouraging. Clinical trials in pediatric patients have yielded similarly promising results, with many more trials of armored and non-armored CAR-T cells in pediatric solid tumors currently active and recruiting.

## Data Availability

No datasets were generated or analysed during the current study.
